# Laboratory investigation of intra-tooth variability in microbial communities from clinically isolated root canal and filling material

**DOI:** 10.1007/s00784-026-07108-y

**Published:** 2026-08-26

**Authors:** Maxi Müller, Ann-Kathrin Weber, Christian Tennert, Lamprini Karygianni, Annette Wittmer, Kirstin Vach, Fabian Cieplik, Ali Al-Ahmad, Konstantin J. Scholz

**Affiliations:** 1 Department of Operative Dentistry and Periodontology, Medical Center & Faculty of Medicine – University of Freiburg, Hugstetterstraße 55, Freiburg, Germany; 2 Department of Restorative, Pediatric and Preventive Dentistry, School of Dental Medicine, University of Bern, Freiburgstrasse 7, 3010 Bern, CH Switzerland; 3 Clinic of Conservative and Preventive Dentistry, Center for Dental Medicine, University of Zurich, Zurich, Switzerland; 4 Institute of Medical Microbiology and Hygiene, Medical Center & Faculty of Medicine, University of Freiburg, Freiburg, Germany; 5 Institute of Medical Biometry and Statistics, Medical Center & Faculty of Medicine, University of Freiburg, Freiburg, Germany; 6 Centre for Data Science, Eberswalde University for Sustainable Development, Eberswalde, Germany

**Keywords:** Endodontic infection, Microbiome, Secondary root canal treatment, Microbial diversity

## Abstract

**Objective:**

Studies on the microbiota of endodontic infections mostly investigate samples from only one, the largest or symptomatic, root canal. This study evaluated the endodontic bacterial composition in multi-rooted teeth by analyzing each root canal (RC) and the root canal filling material (FM) separately, evaluating associations with clinical and radiographic parameters.

**Methods:**

RC and FM samples were collected from 12 teeth. Percussion tests and preoperative radiographs (assessing FM length, homogeneity, periapical index (PAI)) were performed. Culture techniques including calculation of total bacterial count (TBC) and anaerobic/aerobic bacteria followed by identification using MALDI-TOF and 16S rDNA-PCR were conducted. Data were analyzed using mixed linear models (α = 0.05).

**Results:**

Bacterial growth was detected in 27/33 RC and 18/26 FM samples (11/12 teeth). 81 species (41 aerobic/facultative anaerobic/fungi, 40 anaerobic) were identified. There were frequent differences in microbial composition and TBC between root canals of the same tooth, though these were not significant (n.s.). *Dentiradicibacter hellwigii* (gen. nov., sp. nov.) was identified for the first time. In teeth with FM ≥ 3 mm short of the apex and homogenous FM, anaerobic bacteria predominated in RC and FM samples (*p* = 0.0412). Positive percussion sensitivity correlated with higher aerobic/facultative anaerobic TBC (*p* = 0.002). PAI Scores ≥ 4 showed higher anaerobic TBC (*p* = 0.0466).

**Conclusions:**

Microbial composition varies between individual root canals of a tooth and between root canals and filling materials. The TBC was associated with FM quality and clinical parameters.

**Clinical relevance:**

Comprehensive assessment of all root canals is necessary to fully capture the microbial community of endodontic infections.

**Supplementary Information:**

The online version contains supplementary material available at https://doi.org/10.1007/s00784-026-07108-y.

## Introduction

Endodontic infections are characterized by complex microbial communities that develop in root canal systems and periapical tissues following pulp necrosis or reinfection after root canal obturation [32, 36]. Once the pulp becomes infected, the intricate network of dentinal tubules, isthmuses, and accessory canals creates an environment highly favorable for microbial colonization and biofilm formation with a predominance of Gram-positive facultative anaerobes in persistent or secondary infections [1, 35]. In multi-rooted teeth, this complexity is even more pronounced, since multiple canals may communicate with each other or harbor distinct ecological niches themselves, e.g. between the coronal and apical endodontium [41], along the canal length or between mechanically prepared and unprepared areas [16, 37]. Consequently, the microbial diversity and composition within such teeth is expected to be heterogeneous. Previous studies on microbial composition in infected untreated or beforehand obturated root canal infections have provided some insights into the dominant 20–30 bacterial taxa described as the core microbiome of endodontic infections [10, 36]. However, most investigations have relied on samples obtained from a single root canal, usually the main or most easily accessible one, and often correlated microbial findings with periapical radiographs or clinical symptoms alone without distinguishing between the canal systems [17, 32, 36]. Although these studies have advanced our understanding of the microbial ecology in infected root canals, they may underestimate the complexity of endodontic infections in multi-rooted teeth. Differences in microbial composition between individual root canals may not only affect treatment outcomes but also explain persistent apical periodontitis or secondary infections after initial therapy [9, 23].

The role of root canal filling (FM) materials in harboring residual bacteria has gained attention in the context of persistent or secondary endodontic infections [20]. Even when root canals appear adequately obturated radiographically, microorganisms may persist within dentinal tubules or remain trapped within gutta-percha remnants and sealer components, potentially causing polymer degradation of the root filling material [14]. Removal of these materials during retreatment offers the potential to assess the microbial status of previously treated root canals. Similarly, parameters such as the homogeneity or apical extent of canal fillings have been suggested to influence bacterial survival and recolonization, yet few investigations have focused on quantitative analysis of these associations [34]. A better understanding of microbial distribution across different root canals within the same tooth could improve clinical decision-making [34]. Specifically, mapping the microbial loads and compositions in both untreated and retreated canals may reveal why some root canal systems remain symptomatic or develop recurrent infections despite apparent sufficient treatment quality [44]. Additionally, correlating microbial findings with clinical and radiological parameters may provide useful prognostic indicators for case selection and further therapeutic planning [45].

Against this background, the present study was designed to comprehensively evaluate the microbial composition and total bacterial counts (TBC) in multiple canals of the same tooth as well as in the corresponding root canal filling materials. The first null-hypothesis was that the different root canals and filling materials of canal systems within the same tooth do not differ significantly from each other regarding microbial composition and bacterial counts. The second null-hypothesis was that there is no significant influence of clinical and radiographical parameters on microbial composition and total bacterial counts.

## Material and methods

### Ethical considerations and patient inclusion

This study was performed in line with the principles of the Declaration of Helsinki. The design of the present study was approved by the Ethics Committee of the University Medical Center Freiburg (reference number: 140/09). In addition, the quality of the root canal filling was assessed according to the ESE quality guidelines (homogeneity, length, adaptation of filling to root canal walls) [13].

### Assessment of clinical and radiological parameters

Clinical sensitivity to percussion was assessed prior to trepanation of the tooth by tapping the occlusal surface with the blunt handle end of a dental mirror. Patients were asked to indicate whether this caused any subjective discomfort. The parameters of root canal filling sufficiency, homogeneity, and root canal filling length were radiologically evaluated for each tooth by two experienced practitioners, independently of one another. The PAI (Periapical Index according to Ørstavik et al. [22]) was also evaluated by the same evaluators. As the separate roots were not discernible on the two-dimensional radiographic images, the maximum dimension of the apical radiolucency was recorded and applied to all roots of a tooth.

### Sample collection

The tooth to be treated was cleaned with pumice powder and isolated using a rubber dam. The rubber dam and tooth were disinfected by touching with 30% hydrogen peroxide (H_2_O_2_ 30%; J.T.Baker/Avantor, Radnor, USA) and 3% sodium hypochlorite (NaOCl 3%; Hedinger, Stuttgart, Germany). The NaOCl was then neutralized with 5% sodium thiosulphate (Na_2_S_2_O_3_ 5%; Merck, Darmstadt, Germany) for 10 s each. Prior to access cavity preparation, a quality control (QC) was collected from the disinfected rubber dam and tooth surface using a sterile foam pellet to identify possible contamination of the samples.

The access cavity was prepared with diamond burs.

For secondary root canal treatments, root canal fillings were removed down to the apical third without the use of irrigation solutions or solvents using sterile Hedström files (VDW). The filling material of the individual canals was transferred to reduced transport fluid (RTF) and clearly labeled “FM” (= filling material sample).

After removing the root canal filling material or in primary root canal treatments, the individual root canals were explored and instrumented using sterile K-files (ISO 15–35, VDW, München, Germany). Dentin chips and pulp tissue were removed with sterile K-files (ISO 10–25, VDW). Then, 40 μL of a 0.9% sodium chloride solution was added to each canal to prepare it for microbial sampling. in a standard protocol developed in our group in a 2007 study and used in other studies on primary and secondary endodontic infections, adding 40 µl of saline when sampling root canals has been revealed to be sufficient for recovering a high diversity of microbiota in endodontic infections [3, 4, 31, 40]. The samples “RC” (= root canal sample) were then taken from the respective canals with sterile paper points (Antaeos Absorbent Paper Points Taper 0.02 ISO 15, VDW), including pulp tissue. All samples were collected in transport containers, stored in RTF [38], frozen and preserved at -80 °C immediately after sample collection.

### Microbiological analysis

Frozen samples were thawed in a water bath at 37 °C, homogenized using a vortexer (Heidolph Instruments, Schwabach, Germany) and dilution series were prepared on different culture media (Columbia blood agar (CBA); yeast extract-cysteine blood agar according to Beerens [6]). The CBA-plates were incubated at 37 °C and 5–10% CO_2_ for about five days and the yeast cysteine plates were incubated under anaerobic conditions (5% CO_2_, 85% N_2_, 10% H_2_) using airtight pots at 37 °C for ten days.

After incubation, the individual colonies were differentiated according to shape, color, size, consistency, surface and hemolysis behavior. The total bacterial counts were calculated from the number of individual colonies grown and the corresponding dilution level. For individual morphotypes, the mean value was determined across the various dilution levels and the respective bacterial count was calculated accordingly.

For obtaining pure cultures, the individual colonies were each inoculated on CBA (aerobes and facultative anaerobes; incubation at 37 °C for at least 24 h in a CO_2_ cabinet) and yeast cysteine blood agar plates (anaerobes; incubation at 37 °C for 2 days in the anaerobic pots).

MALDI-TOF–MS (matrix assisted laser desorption—time of flight mass spectrometry) was used to differentiate microbes previously obtained in pure cultures on a MALDI Biotyper Microflex LT (Bruker Daltonik GmbH, Bremen, Germany), following the method described in previous studies [5, 29]. If no reliable identification could be achieved after several runs, further investigation methods were used for differentiation:

For aerobic and facultative anaerobic bacteria, Gram stainings were prepared and examined morphologically using light microscopes. The samples were classified into Gram-positive or Gram-negative species and classification to cocci or rods. Additionally, enzyme tests (Anaerobe Laboratory Manual, The Virginia Polytechnic Institute and State University Anaerobe Laboratory, Blacksburg, Virginia 1977); e.g. catalase activity of particularly aerobic bacteria e.g., staphylococci and some *Actinomyces* spp., were applied.

For anaerobic bacteria, spot indole tests (DMACA Indole assay, Becton, Dickinson and Company, Sparks, USA) for tryptophanase detection and CO_2_ control plates to determine the presence of facultative or obligate anaerobes were performed.

To verify results of the microbiological test methods, DNA was extracted from isolated individual colonies, a polymerase chain reaction using the following primers were used: RT16U6 (reverse: 5' ATT GTA GCA CGT GTG T[A/C]G CCC -3') and TP16U1 (forward: 5' AGA GTT TGA TC[C/A] TGG CTC AG -3'). The amplification products (1233 bp) were sent for sequencing (GATC Biotech AG, Constance, Germany) [27].

The base sequences obtained were then compared in the international NCBI database (http://www.ncbi.nlm.nih.gov/BLAS) or SepsiTest™ BLAST (Molzym, Bremen, Germany), and assigned to the respective species or genera. If the match was > 97.5%, the detection of the respective taxon was confirmed.

### Statistical analysis

The mean (standard deviation) was calculated for descriptive analysis. For this purpose, the canals of a tooth were, according to their typical size, divided into main (lower molars: distal canal; upper molars: palatal canal; labeled as canal 1) and secondary canals (labeled as canal 2 or 3). The individual species as well as the total aerobic/facultative anaerobic and anaerobic bacterial count and diversity were taken into account. For each canal or root filling material, the fraction of each species referring to the total bacterial count was presented, respectively.

For an initial analysis, the values of the secondary channels were combined, as two secondary channels were not always present. To analyze differences of the bacterial residues between the filling material sample and the root canal sample, the values per filling and per cleaned root canal were averaged. Afterwards a paired t-test was used for comparison. For bacterial groups in which no bacteria were detected in several teeth, the Friedman test was used.

Mixed linear models were used to investigate associations between the microbiological analyses and the clinical parameters. The statistical analysis was performed using STATA 19.0. (StataCorp LLC, College Station, Texas, USA). The level of significance was set to α = 0.05.

## Results

The present study included 10 patients (8 female, 2 male) with 12 teeth (33 root canals) in need of primary (2 teeth) or secondary (10 teeth) endodontic treatment. In this study, according to self-report of the patients, the estimated age of the root canal fillings needing revision treatment was between 2 and 15 years.

The clinical and radiographic parameters and diagnoses of each tooth are listed in Table 1.

**Table 1:** Overview of clinical and radiographic parameters and diagnoses (RC = root canal; FM = root canal filling material)

patient	tooth	type of tooth	no. of RC	endodontic/ periapical diagnosis	sensitivity to		primary or secondary infection	homo-geneity of root canal filling	max. distance from root filling to apex in mm	apical lesion (PAI)	commentary on apical lesion
					cold	per-cussion					
1	1	25	2	endo-periodontal lesion	+	-	primary	/	/	3	apical lesion and vertical bony defect
2	2	24	2	acute apical periodontitis	-	+	secondary	no	2	1	/
3	3	26	3	no periapcal pathology visible	-	-	secondary	yes	3	1	/
4	4	46	3	chronic apical periodontitis	-	-	secondary	no	3	5	m root: 2-3 mm
5	5	16	3	chronic apical periodontitis	-	+	secondary	no	0	4	mb root: 2-3 mm
6	6	17	3	chronic apical periodontitis	-	-	secondary	no	3	4	m root: extended diffuse lesion
7	7	47	3	chronic apical periodontitis with secondary acute progression	-	+	secondary	yes	3	5	m and d root: 3 mm
8	8	16	3	chronic apical periodontitis	-	+	secondary	no	2	3	d + p root
	9	26	3	chronic apical periodontitis	-	+	secondary	no	3	3	p root
9	10	24	3	endo-periodontal lesions	-	-	secondary	No	0	2	b + p root (no FM material in 1 b RC)
	11	26	3	chronic apical periodontitis	-	-	secondary	yes	0	4	p root: 1 mm
10	12	25	2	chronic apical periodontitis	-	-	primary	/	/	3	2 mm

Microbial growth was detected in 11 of 12 teeth (92%). A total of 81 different bacterial species were detected. Further, *Candida* spp. could be isolated from one root canal sample of a secondary infection with symptoms of acute apical periodontitis.

When looking at the examined teeth as individual cases, some species were only detected in one of the two or three root canals (teeth with two root canals: average of 54.8% of species only in one canal; teeth with three root canals: average of 71.7% of species only in one canal). The paired t-tests and Friedman tests performed did not reveal any significant differences in microbial distribution between the canals of a tooth.

An overview of the total bacterial counts (TBC), all detected species per tooth, number of species identified per tooth and per sample (RC, FM) and their percentage distribution can be found in Table 2. The supplementary data contain lists of the bacteria detected, categorized according to aerobic/facultative anaerobic or anaerobic bacteria (Supplementary Table 1) and primary and secondary infections (Supplementary Table 2).

**Table 2:** Overview of the total bacterial counts (TBC) and number of species found per tooth, per individual root canals (RC) and individual root canal filling material (FM)

patient	tooth	type of tooth	no. of RC	TBC per tooth (all RC + FM) in CFU/ml	no. of species per tooth (RC and FM)	species	RC/FM Name	TBC per RC or FM in CFU/ml	% of TBC of RC/FM	no. of species per RC/FM
1	1	25	2	1.3 × 10^8^	5	*S. oralis, M. osloensis, P. nigrescens, F. nucleatum, O. uli*	RC1	9.0 × 10^7^	67.9%	5
							RC2	4.3 × 10^7^	32.1%	4
2	2	24	2	2.9 × 10^6^	18	*S. oralis, S. mitis, A. odontolyticus, A. naeslundii, R. dentocariosa, L. casei/paracasei, L. fermentum, N. elongata, N. flavescens, C. ochracea, C. albicans, F. nucleatum, V. parvula, A. geminatus, O. uli, O. profusa, P. denticolens, P. acnes*	RC1	1.0 × 10^5^	10.5%	1
							RC2	8.6 × 10^5^	89.5%	10
							FM1	1.2 × 10^6^	59.2%	6
							FM2	8.1 × 10^5^	40.8%	7
3	3	26	3	0	0	/	RC1	0	0	0
							RC2	0	0	0
							RC3	0	0	0
							FM1	0	0	0
							FM2	0	0	0
							FM3	0	0	0
4	4	46	3	2.0 × 10^7^	14	*S. oralis, S. para-/sanguinis, S. constellatus, E. faecalis, R. dentocariosa, R. ornithinolytica, P. gingivalis, F. nucleatum, D. invisus, S. exigua, A.rimae, B. extructa, O. uli, P. micra*	RC1—mb	7.0 × 10^4^	2.0%	6
							RC2—d	2.3 × 10^5^	6.5%	8
							RC3—ml	3.2 × 10^6^	91.5%	9
							FM1—mb	7.5 × 10^4^	0.5%	4
							FM2—d	1.0 × 10^4^	0.1%	5
							FM3—ml	1.6 × 10^7^	99.5%	8
5	5	16	3	5.6 × 10^6^	24	*S. oralis, S. anginosus, S. constellatus, S. mutans, E. faecalis, A. oris, A. odontolyticus, A. naeslundii, A. israelii, Actinomyces* spp.*, R. mucilaginosa, R. dentocariosa, P. gingivalis, black-pigmented bacteria, F. nucleatum, V. parvula, D. invisus, S. exigua, P. alactolyticus, B. dentium, A. rimae, O. uli, P. micra, P. acnes*	RC1—mb	5.1 × 10^5^	18.9%	6
							RC2—p	2.4 × 10^5^	8.8%	8
							RC3—db	2.0 × 10^6^	72.3%	11
							FM1—mb	2.4 × 10^6^	84.5%	8
							FM2—p	4.1 × 10^5^	14.3%	11
							FM3—db	3.2 × 10^4^	1.1%	5
6	6	17	3	2.0 × 10^3^	1	*P. acnes*	RC1—mb	2.0 × 10^3^	100.0%	1
							RC2—p	0	0	0
							RC3—db	0	0	0
							FM1—mb	0	0	0
							FM2—p	0	0	0
							FM3—db	0	0	0
7	7	47	3	3.3 × 10^7^	34	*S. oralis, S. mitis, S. para-/sanguinis, S. gordonii, S. constellatus, G. haemolysans, A. oris, A. odontolyticus, A. naeslundii, R. mucilaginosa, R. dentocariosa, R. aeria, Neisseria* sp.*, C. hominis, P. nigrescens, A. tannerae, P. tannerae, Bacteroides* spp.*, F. nucleatum, F. periodonticum, C. rectus, T. forsythia, Veillonella* spp.*, D. invisus, A. geminatus, S. exigua, M. timidum, F. alocis, E. infirmum, A. rimae, Atopobium* sp.*, O. uli, O. profusa, P. micra*	RC1—mb	6.0 × 10^6^	88.3%	14
							RC2—d	4.1 × 10^5^	6.0%	8
							RC3- ml	3.8 × 10^5^	5.7%	8
							FM1—mb	1.4 × 10^7^	52.9%	9
							FM2—d	2.0 × 10^6^	7.6%	12
							FM3—ml	1.0 × 10^7^	39.5%	14
8	8	16	3	6.0 × 10^3^	2	*P. acnes, R. dentocariosa*	RC1—mb	0	0	0
							RC2—p	0	0	0
							RC3- db	3.0 × 10^3^	100.0%	1
							FM1—mb	0	0	0
							FM2—p	3.0 × 10^3^	100.0%	1
							FM3—db	0	0	0
	9	26	3	4.6 × 10^7^	14	*G. morbillorum, G. adiacens, E. faecalis, A. lingnae, C* *gingivalis, P. oris, F. nucleatum, C. showae, M. micronuciformis, F. alocis, S. satelles, L. saburreum,* *O. profusa, P. micra*	RC1—mb	3.6 × 10^5^	1.2%	4
							RC2—db	3.0 × 10^7^	98.3%	7
							RC3- ml	1.7 × 10^5^	0.5%	6
							FM1—mb	4.6 × 10^6^	31.1%	8
							FM2—db	1.5 × 10^6^	10.3%	5
							FM3—ml	8.7 × 10^6^	58.6%	7
9	10	24	3	2.7 × 10^4^	11	*S. anginosus, S. intermedius, G. adiacens, A. meyeri, Dentiradicibacter hellwigii, V. parvula, D. invisus, S. exigua, E. catenaformis, A. rimae, P. micra*	RC1—b1	2.0 × 10^3^	7.4%	2
							RC2—b2	1.0 × 10^4^	37.0%	4
							RC3—p	1.5 × 10^4^	55.6%	7
	11	26	3	2.2 × 10^6^	22	*S. oralis, S. salivarius, S. anginosus, S. constellatus, S. intermedius, G. adiacens, E. durans, A. johnsonii, R. aeria, N. macacae/mucosa, N. oralis, C. ochracea, C. granulosa, K. oralis, P. gingivalis, F. nucleatum, B. wadsworthia, V. parvula, E. yurii, O. profusa, P. micra, P. avidum*	RC1—mb	1.6 × 10^6^	87.9%	9
							RC2—p	5.6 × 10^4^	3.1%	11
							RC3- db	1.6 × 10^5^	9.0%	6
							FM1—mb	3.2 × 10^5^	81.0%	6
							FM2—p	6.0 × 10^3^	1.5%	1
							FM3—db	6.8 × 10^4^	17.5%	4
10	12	25	2	4.9 × 10^9^	22	*S. oralis, S. anginosus, S. constellatus, A. oris, A. naeslundii, R. mucilaginosa, R. dentocariosa, N. bacilliformis, C. granulosa, P. gingivalis, P. intermedia, F. nucleatum, T. forsythia, V. parvula, D. pneumosintes, A. geminatus, S. exigua, B. dentium, F. alocis, E. yurii, O. uli, P. micra*	RC1 -b	2.4 × 10^8^	4.9%	14
							RC2—p	4.7 × 10^9^	95.1%	18

### Primary infections

The total aerobic/facultative anaerobic bacterial count of the teeth ranged from 6.0 × 10^3^ CFU/ml to 1.2 × 10^8^ CFU/ml, with a mean value of 5.8 × 10^7^ ± 8.2 × 10^7^ CFU/ml (2.9 × 10^7^ ± 5.1 × 10^7^ CFU/ml per root canal). The total anaerobic bacterial count was between 1.3 × 10^8^ CFU/ml and 4.8 × 10^9^ CFU/ml, with a mean value of 2.5 × 10^9^ ± 3.3 × 10^9^ CFU/ml (1.2 × 10^9^ ± 2.2 × 10^9^ CFU/ml per root canal). Quantitatively, anaerobes therefore predominated in the primary infections.

Microorganisms were detected in both cases (100%) with primary infection and both cases showed polymicrobial growth. A total of 24 species were identified with a mean value of 13.5 ± 12.0 species per tooth and 10.3 ± 6.8 species per root canal. Looking at the entire microbial flora of primary infections, 10 aerobes and facultative anaerobes (42%) and 14 anaerobes (58%) were identified.

(For further details regarding the total bacterial counts and species found see Tables 2 and 3, Supplementary Fig. 1, 2, 3, 4 and 5).

**Table 3:** Mean total bacterial counts (CFU/mL) separated in to primary and secondary infections an aerobes and anaerobes per tooth, per root

	TBC mean (CFU/mL)					
	tooth all	tooth aerobic/ facultative anaerobic	tooth anaerobic	Root all	root aerobic/ facultative anaerobic	root anaerobic
primary infections	2.5 × 10^9^ ± 2.3 × 10^9^	5.8 × 10^7^ ± 8.2 × 10^7^	2.5 × 10^9^ ± 3.3 × 10^9^	1.3 × 10^9^ ± 1.5 × 10^9^	2.9 × 10^7^ ± 5.1 × 10^7^	1.2 × 10^9^ ± 2.2 × 10^9^
secondary infections root canals	2.8 × 10^6^ ± 7.3 × 10^6^	3.7 × 10^5^ ± 6.6 × 10^5^	5.3 × 10⁶ ± 1.0 × 10^7^	9.0 × 10^5^ ± 8.7 × 10^4^	1.3 × 10^5^ ± 3.6 × 10^5^	1.7 × 10^6^ ± 6,0 × 10^6^
root canal fillings	4.1 × 10^6^ ± 7.1 × 10^6^	1.2 × 10^6^ ± 2.2 × 10^6^	6.9 × 10^6^ ± 9.2 × 10^6^	1.4 × 10^6^ ± 3.4 × 10^6^	4.2 × 10^5^ ± 8.9 × 10^5^	2.3 × 10^6^ ± 4.6 × 10^6^

### Secondary infections

For secondary infections, the total aerobic/facultative anaerobic bacterial count of the root canal samples (RC) ranged from 2.0 × 10^3^ CFU/ml to 2.2 × 10^6^ CFU/ml with a mean value of 3.7 × 10^5^ ± 6.6 × 10^5^ CFU/ml per tooth (1.3 × 10^5^ ± 3.6 × 10^5^ CFU/ml per canal). The total anaerobic bacterial count varied between below the detection limit for one tooth and up to 3.0 × 10⁷ CFU/ml, with a mean value of 5.3 × 10⁶ ± 1.0 × 10^7^ CFU/ml (1.7 × 10⁶ ± 6.0 × 10^6^ CFU/ml per canal). In the root canal filling material samples (FM), total aerobic/facultative anaerobic bacterial counts of 0.0 CFU/ml to 6.3 × 10^6^ CFU/ml were found, with a mean value of 1.2 × 10^6^ ± 2.2 × 10^6^ CFU/ml per tooth (4.2 × 10^5^ ± 8.9 × 10^5^ CFU/ml per canal). The revealed total anaerobic bacterial count in FM-samples ranged from 0.0 CFU/ml to 2.5 × 10^7^ CFU/ml, with a mean value of 6.9 × 10^6^ ± 9.2 × 10^6^ CFU/ml (2.3 × 10^6^ ± 4.6 × 10^6^ CFU/ml per canal).

Anaerobes therefore also dominated the secondary infections. In the percentage distribution of the total bacterial count, there are significant differences between the individual canals in some cases. For example, in each case, when only root canal or root filling material samples are considered, more than 50% of the microorganisms were found in only one canal.

The secondary infections showed microbial growth in 9 out of 10 cases (90%). A total of 76 different bacterial species and *Candida albicans* were present. A total of 62 different taxa were detected in the root canal samples (RC) with a mean value of 10.4 ± 8.0 species per tooth and 5.1 ± 4.2 species per canal.

The root canal filling material samples (FM) contained a total of 53 different taxa. Up to 14 species were found in each case, or up to 27 per tooth. The average was 9.8 ± 8.9 taxa per tooth and 4.7 ± 4.2 taxa per canal.

In 6 patients, some taxa were present in both samples. 0–14 different species were detected per canal and 1–22 different species per tooth. The total bacterial flora can be divided into 38 aerobes and facultative anaerobes (50%) as well as 38 anaerobes (50%). No bacteria could be detected in one tooth (10%). In patient 6 (10%), one species was identified in only one root canal. In patient 8, tooth 16 (10%), only one species was found in both the root canal sample (RC) and the filling material (FM). All other cases (70%) showed a polymicrobial flora.

The taxon most frequently detected in secondary infections was *Enterococcus faecalis*, both in seven root canals (26.9%) and root fillings (30.4%), as well as *Olsenella uli* in seven root canals (26.9%) and six root fillings (26.1%).

**Fig. 1:**
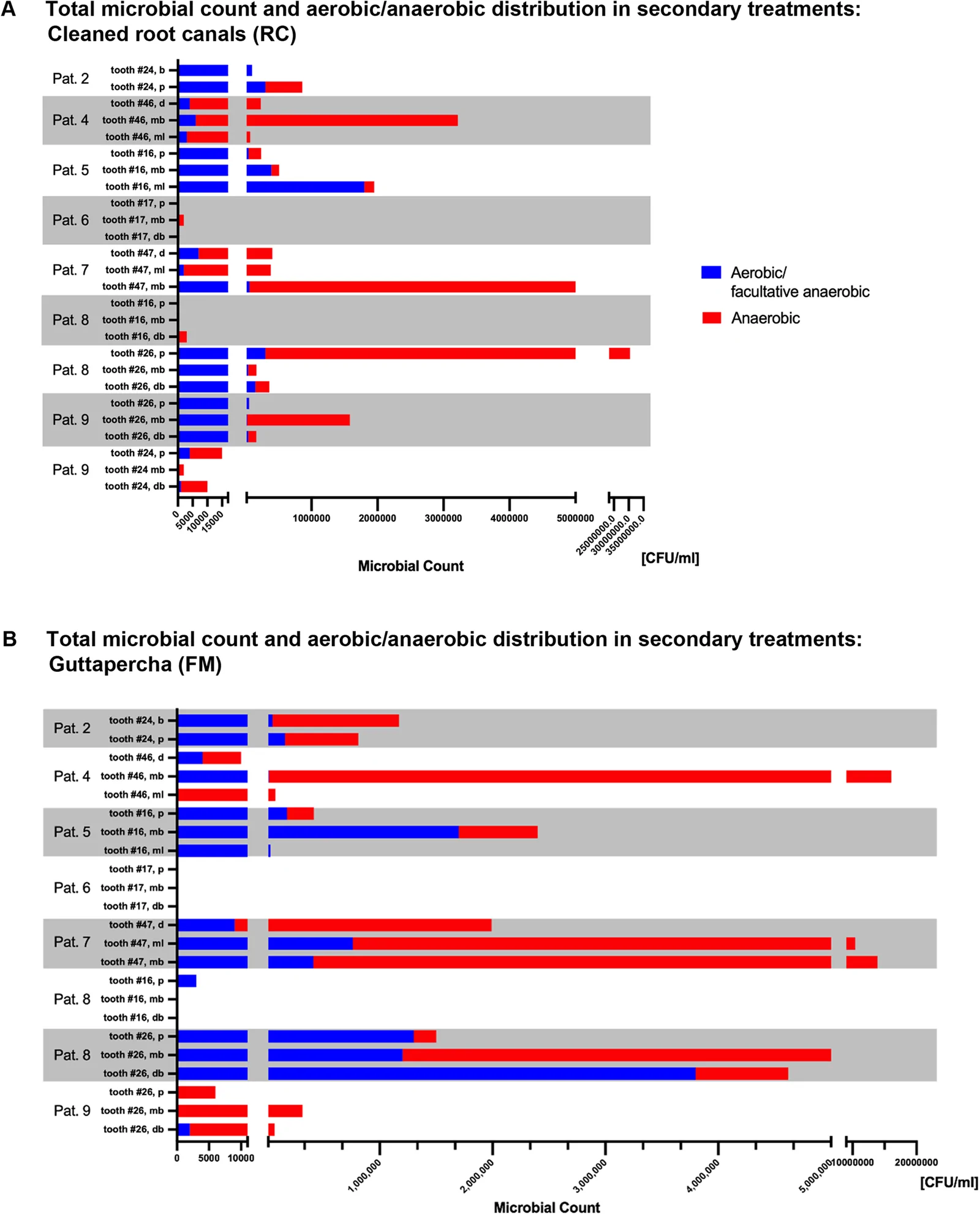
**A** Total microbial count (in CFU/ml) and aerobic/facultative anaerobic and anaerobic distribution in sample of secondary endodontic treatments; listed per root canal. **B** Total microbial count (in CFU/ml) and aerobic/facultative anaerobic and anaerobic distribution in samples of root canal filling materials of secondary endodontic treatments; listed per root canal

*Dentiradicibacter hellwigii*, a novel species of a new genus in the class *Betaproteobacteri*a (type strain: WK13^T^ (= DSM 112713^ T^ = NCTC 14938^ T^) was isolated from a secondary infected root canal in the human oral cavity for the first time during this study in Proband 9, Tooth 10 [8]. It grew poorly in the presence of other bacterial colonies on a yeast extract-cysteine blood agar, and MALDI-TOF failed to identify it. It requires nursing bacteria, such as *Prevotella intermedia*, *Capnocytophaga granulosa*, and *Capnocytophaga sputigena*, in order to grow on agar plates. Following co-cultivation with these bacteria, sufficient material was obtained to sequence the 16S rRNA gene. This revealed a new species, which was ultimately certified by the National Collection of Type Cultures in Germany and the United Kingdom.

(For further details regarding total bacterial counts and the species found see Tables 2 and 3, Fig. 1A + B; Supplementary Data Figs. 3, 4, and 5).

### Influence of clinical and radiographic parameters on microbial composition

#### Distance of root canal filling to the apex

**Fig. 2:**
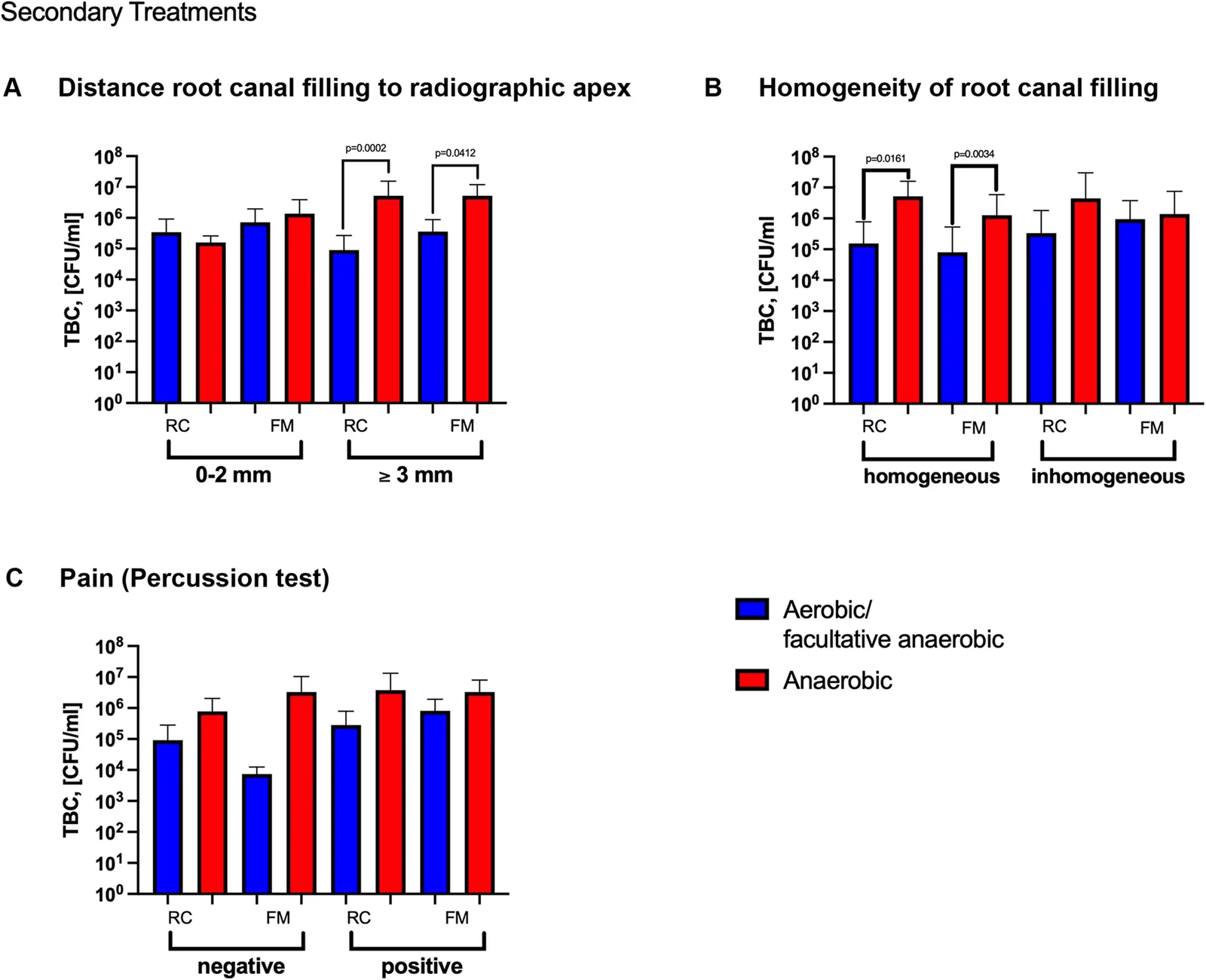
**A** Total bacterial count of anaerobic and aerobic/facultative anaerobic bacteria depending on root filling length. **B** Total bacteria count of aerobic/facultative anaerobic bacteria depending on root filling homogeneity. **C** Total bacterial count of anaerobic and aerobic/facultative anaerobic bacteria depending on pain on percussion test

The aerobic/facultative anaerobic and anaerobic TBC showed associations with the distance to the apex in RC samples (aerobic/facultative anaerobic: *p* = 0.001, anaerobic: *p* = 0.004) but not in FM samples. While teeth with root fillings with a distance of 0—2 mm to the apex showed no significant differences in the proportions of aerobes and anaerobes; in teeth with root fillings with ≥ 3 mm distance to the apex, anaerobic bacteria predominated in RC (*p* = 0.002) and FM (*p* = 0.0412) (see Fig. 2A).

#### Homogeneity of root canal filling

In secondary treatments with homogenous root canal fillings, anaerobic TBC was significantly higher than aerobic/facultative anaerobic TBC in RC (*p* = 0.0161) and FM (*p* = 0.0034) samples; but not in cases with inhomogeneous root canal fillings (See Fig. 2B).

#### Sensitivity to percussion

In the samples from the cleaned canals, no significant differences between aerobic/facultative anaerobic and anaerobic TBC were found in teeth with and without percussion sensitivity.

However, in the root canal filling samples a positive sensitivity to percussion was associated with a higher aerobic/facultative anaerobic TBC in FM samples (*p* = 0.002). (See Fig. 2C).

#### PAI

This study found no correlation between the PAI Scores 1–3 and the total bacterial count of aerobic/facultative anaerobic and anaerobic bacteria in RC or FM. Only for PAI Scores 4–5 anaerobic TBC was higher than the aerobic/facultative anaerobic TBC in RC samples (*p* = 0.0466).

## Discussion

The present study aimed to investigate the endodontic microbiota in multi-rooted teeth by separately analyzing all root canals and corresponding root canal filling materials and showed greater differences between the microbial composition in different root canals of a single tooth than previously assumed from other studies. In some cases, bacterial growth was observed in only one root canal, while cultivable bacteria were only detected in the root canal filling of another canal. Furthermore, there were differences between total bacterial counts as well as the microbial composition between the root canals and the root filling materials inside the respective canals by tendency without reaching the level of statistical significance, which led to a partial rejection of the first null hypothesis. Additionally, root canals of primary infections also showed higher total bacterial counts than root canals following retreatment and root canal fillings of secondary infections. The second null-hypothesis could be rejected, as the total bacterial count of aerobic/facultative anaerobic and anaerobic bacteria influenced were associated with clinical and radiographic parameters in terms of a significantly higher anaerobic TBC in radiographically too short and homogeneous root canal fillings as well as in RC samples of teeth with a PAI of 4 or 5. Only in the root canal filling samples of teeth with a positive sensitivity to percussion a significantly higher aerobic/facultative anaerobic TBC could be found.

Numerous studies have investigated microbial colonization of the root canal system. They focused on a wide range of topics, including differences between primary and secondary treatments, the coronal and apical sections, correlations with clinical and radiological findings, and the effectiveness of disinfection protocols [18, 33, 36]. But these previous studies almost always only examined samples from a single root canal, usually the largest canal or the one with radiographic apical radiolucency, to determine the microbial flora of multi-rooted teeth [15]. This study is the first to evaluate the microbial composition of endodontic infections for each root canal of a tooth individually using a combined approach of culture techniques and subsequent identification of isolated microorganisms using different methods.

To the best knowledge of the authors, only one other study included more than one sample per tooth when investigating the endodontic microbiota. Chugal et al. took samples of different root canals of a single tooth, but only from three teeth and investigated the microbiota residing specifically at the apical portion of extracted, primarily or secondarily treated root canals using PCR and DNA sequencing [12]. The microbiota differed slightly between those root canals, where the tooth with a secondary infection showed a greater variation between the root canals of a tooth than the one with a primary infection. Gomes et al. investigated the root canal microbiota of primary and secondary infections in an in vivo study [15]. They took samples from one root canal per tooth and analyzed them using culture technique and came to similar results as presented here [15].

Other studies comparing the sulcus and root canal system using 16S rRNA sequencing found that *Shuttleworthella satelles*, *Olsenella uli*, *Dialister invisus*, *Massilia timonae*, and *Klebsiella pneumoniae* were present in sulcus and root canal system, suggesting microbial migration. Although the authors included control teeth for the sulcus samples and samples from 30 teeth in 25 patients, they did not separate vestibular and lingual sulci or, unlike our study, individual canal samples to investigate this migration more precisely [25].

In a similar experimental design including 10 teeth with primary and secondary endoodontic infection each, the same group observed at the species level that *Neisseria macacae*, *Streptococcus gordonii*, *Bifidobacterium dentium*, *Stomatobaculum longum*, and *Schaalia odontolytica* were significantly increased in root canals with secondary persistent endodontic infection compared to primary endodontic infection [24].

When comparing caries and apical periodontitis via next-generation sequencing, genera such as *Acinetobacter*, *Dialister*, *Lawsonella*, *Phocaeicola*, *Mogibacterium*, *Pyramidobacter*, and *Parvimonas* were more abundant in canal samples than in biofilms from both caries-free surfaces and dentin caries [39]. As the root canal samples differed significantly from both biofilms, which themselves did not differ significantly, it can be inferred that bacterial penetration into the pulp system creates a new niche that strongly influences the microbial composition. Moreover, the present study showed differences in microbial composition within canal systems.

In the periapical environment of infected teeth, these microorganisms directly interact with and are influenced by the host immune response [43]. In this study, the microbiological composition only showed a predominance of anaerobic bacteria for PAI Scores 4–5. But radiographically, only two-dimensional orthoradial images were taken. With these, only a very limited assessment of the actual size of the apical lesions and possible additional, non-instrumented root canals was possible. Nevertheless, individual periapical radiographs are still the clinical standard for radiographic imaging during endodontic treatment, aiming for an x-ray exposure as low as reasonably achievable. In another study that analyzed the coronal and root canal microbiota in apical periodontitis with different PAI scores using 16S rRNA sequencing, most of the bacterial species found to be increased in the PAI groups were strict anaerobes. Moreover, apical periodontitis with higher PAI values was associated with a diminished microbial diversity [41]. In subsequent studies, three-dimensional imaging distinguishing between the particular separate root canal systems could provide a more accurate assessment [30]. In further studies, consideration should be given to using three-dimensional imaging (small-volume CBCT) rather than the PAI-Index, which is based on less accurate two-dimensional imaging, in order to be able to make clear statements about the apical response of individual canals.

To avoid cross-contamination between root canals, it was necessary to conduct the removal of the root filling materials without any liquid or lubricant. However, after completion of the sampling, regular chemical disinfection protocols involving NaOCl and EDTA were applied in order to complete the root canal preparation *lege artis.* Other methods for analysis which involve pulverization of teeth following extraction may result in a lower detection limit for the bacteria [2, 42], but this method is obviously more invasive and thus only available for extracted teeth.

Despite this elaborate sampling protocol used in the present study, samples from the individual root canals could not be separated into coronal and apical sections [2]. The literature indicates that bacteria can be present in the coronal portion of the root due to coronal insufficiencies, even when there are no detectable bacteria in the apical root section and periapical healing has occurred. [28]. A suchlike approach is not feasible in in vivo protocols, but further studies could differentiate the samples in this respect even further in order to determine more precisely which bacteria are involved in the infection and inflammatory response.

One limitation of culture-based methods is their inability to detect fastidious anaerobes and bacteria in the viable but non-culturable state [19]. These are often undetectable using these techniques, which may explain why some root canals in this study revealed no colony-forming units. Meanwhile, the use of a diverse array of culture media (culturomics) has been suggested to fulfil the nutritional requirements of as many oral microorganisms as possible, in order to detect and isolate them from their natural oral niches [7].

Culture-independent molecular techniques have expanded the spectrum of detectable microorganisms in recent years. While traditional culture technique provides quantitative data on viable bacteria, subsequent identification of the isolated colonies can be achieved by MALDI-TOF–MS (matrix-assisted laser desorption/ionization – time-of-flight mass spectrometry). In contrast, molecular approaches such as 16S rRNA sequencing enable the identification of both culturable and, importantly, unculturable species, the latter being underrepresented in studies relying solely on culture-based techniques [7, 19].

MALDI-TOF–MS, which was combined with culture technique in the present study, is characterized by lower material costs per sample compared to sequencing and, due to the comparison with data sets of known bacterial species, by rapid sample analysis with a high degree of certainty. However, reliable identification is often not possible even after several runs if there are insufficient database entries [26]. Therefore, in these cases, bacteria were identified by sequencing of the 16S rRNA gene. Most interestingly, the techniques used in the present study resulted in the culturing and identification of a novel genus, *Dentiradicibacter hellwigii*, demonstrating the advantages of sampling all root canals and combining different microbiological methods [8]. Given the fact that only about half of oral bacteria have ever been cultured until now despite being detected by sequencing methods, it may be possible in future studies to culture additional bacteria from endodontic infections that have so far been considered unculturable [7, 19].

A limitation of the present study is the small sample size of 10 patients or 12 teeth, respectively, but with 33 canals investigated separately it had a similar sample size as comparable studies [24, 39]. Given the time-intensive culture technique the dataset is considerable and provides a solid basis for future studies with larger sample size.

Nevertheless, our findings should be interpreted with a certain caution as the temporal dynamics of infection could not be assessed due to the one sampling time point. Consequently, it is plausible that some bacterial species relevant to the initial pathogenesis of endodontic infections may have been no longer detectable at the time of sampling [36]. Endodontic infections are mixed infections primarily in the form of biofilms, which both emphasizes the importance of a sufficient chemical and mechanical disinfection protocol [44]. From an endodontic-therapeutic perspective, it would be desirable to tailor disinfection protocols or strategies for host immune modulation more specifically to the microbial composition present in the root canal system. Beyond optimizing treatment, such an approach may also provide insights into systemic health, considering the complex interactions between the oral microbiome and the host [44]. In the future, approaches that enable rapid, chairside identification of relevant bacterial species—or the use of microbial signatures as proxies for patient-related factors such as age, history of antibiotic therapy, systemic conditions, or combined periodontal-endodontic involvement—would represent a significant step forward in precision endodontics.

For further studies, it can be concluded from the results of this study that the collection of samples from all root canals in multi-rooted teeth, as well as root filling material in secondary treatment cases, can provide more accurate and comprehensive information about the microbial colonization of the root canal system complexity of the bacterial infection.

Although partial retreatment of only singular root canal systems leaving the radiographically sufficient obturated canals untouched may be considered in certain situations for reasons of cost-effectiveness, this strategy should be viewed with caution [11]. Arguments for the sole revision of individual canals of a tooth and the use of targeted systemic antibiotics should be weighed up and evaluated critically on the basis of this study. Our findings, which demonstrate that bacterial presence was consistently detected across all canal systems, suggest that partial revision of single root canals is unlikely to be adequate. Instead, comprehensive retreatment appears to be the more evidence-based and clinically reliable approach in order to effectively address the persistent microbial burden [21]. Further studies could deepen the understanding of the correlations with clinical symptoms and radiologic parameters through a larger number of cases and the inclusion of further clinical and radiographic parameters or three-dimensional x-ray diagnostic in terms of cone-beam CT.

## Conclusions

The present study observed only non-significant statistical variations in microbial communities across different root canals and root filling material samples within the same teeth requiring primary or secondary endodontic treatment, which may mainly be attributed to the small sample size.

The findings indicated that root canal filling quality, clinical symptoms, e.g. sensitivity to percussion, and the presence of periapical lesions were associated with the microbial composition and total bacterial counts. These findings highlight the need for a deeper understanding of the complexity of the microbiota in multi-rooted teeth and support the development of further diagnostic and therapeutic strategies in endodontics.

## Supplementary Information

Below is the link to the electronic supplementary material.

Supplementary file1 (DOCX 2379 KB)

## Data Availability

No datasets were generated or analysed during the current study.
