# Supplementary material for: Laboratory investigation of intra-tooth variability in microbial communities from clinically isolated root canal and filling material
Source: Clin Oral Investig. 2026 Aug 26;30(9):419. doi: 10.1007/s00784-026-07108-y (PMC13518401; doi:10.1007/s00784-026-07108-y)
Supplement: Supplementary file 1 [file 784_2026_7108_MOESM1_ESM.docx]

***Supplementary data***

*Supplementary Tab. 1: Overview of frequency of occurrence of microorganisms*

*In relation to the 12 cases examined and separated by type of sample; summarized by aerobic/facultative anaerobic vs. anaerobic microorganisms (aerobes summarized in groups for clarity; pathogenic anaerobes listed individually); SS = saliva sample for quality control*

| **Aerobes** | **Anaerobes** |
| --- | --- |
| - *Mitis*-group (27x in 8 cases; 67%; 13x RC, 7x, 7x SS) - *Salivarius*-group (2x in 2 cases; 17%; 1x RC, 1x SS) - *Anginosus*-group (20x in 6 cases; 50%; 12x RC, 6x FM, 2x SS) - *Streptococcus mutans* 3x in 1 case; 8%; 2x RC, 1x FM) - *Gemella/Granulicatella*-group (11x in 5 cases; 42%; 6x RC, 3x FM, 2x SS) - *Enterococci*-group (15x in 4 cases; 33%; 8x RC, 7x FM) - *Actinomyces*-group (21x in 8 cases; 67%; 8x RC, 8x FM, 5x SS) - *Rothia/Lactobacillus*-group (21x in 7 cases; 58%; 8x RC, 6x FM, 7x SS) - *Neisseria*-group (12x in 6 cases; 50%; 7x RC, 1x FM, 4x SS) - *Capnocytophaga*-group (7x in 4 cases; 33%; 5x RC, 1x FM, 1x SS) - *HACEK*-group (3x in 2 cases; 17%; 2x RC, 1x FM) - *Water bacteria* (2x in 1 case; 8%; 1x RC, 1x FM) - *Candida* spp. (1x in 1 case; 8%; 1x RC) | - *Propionibacteria* (8x in 5 cases; 42%; 5x RC, 3x FM) - *Porphyromonas gingivalis* (10x in 4 cases; 33%; 7x RC, 3x FM) - *Prevotella intermedia* (1x in 1 case; 8%; 1x RC) - *Prevotella nigrescens* (5x in 3 cases 25%; 2x RC, 2x FM, 1x SS) - *Alloprevotella tannerae* (2x in 1 case; 8%; 2x RC) - *Prevotella tannerea* (1x in 1 case; 8%; 1x RC) - *Black-pigmented bacteria* (3x in 1 case; 8%; 2x RC, 1x FM) - *Prevotella oris* (3x in 1 case; 8%; 3x RC) - *Bacteroides* spp. (1x in 1 case; 8%; 1x FM) - *Fusobacterium nucleatum* (22x in 9 cases; 75%; 11x RC, 6x FM, 5x SS) - *Fusobacterium periodonticum* (1x in 1 case; 8%; 1x FM) - *Campylobacter rectus* (1x in 1 case; 8%; 1x FM) - *Campylobacter showae* (3x in 1 case; 8%; 1x RC, 2x FM) - *Tannerella forsythia* (3x in 2 cases; 17%; 2x RC, 1x FM) - *Bilophila wadsworthia* (4x in 1 case; 8%; 2x RC, 2x FM) - *Dentiradicibacter hellwigii* (1x in 1 case; 8%; 1x RC) - *Veillonella parvula* (12x in 7 cases; 58%; 4x RC, 3x FM, 5x SS) - *Veillonella* spp. (2x in 2 cases; 17%; 1x FM, 1x SS) - *Dialister invisus* (14x in 4 cases; 42%; 8x RC, 5x FM, 1x SS) - *Dialister pneumosintes* (1x in 1 case; 8%; 1x RC) - *Anaeroglobus geminatus* (5x in 3 cases; 25%; 3x RC, 2x FM) - *Megasphaera micronuciformis* (2x in 2 cases; 17%; 1x FM, 1x SS) - *Slackia exigua* (12x in 5 cases; 42%; 9x RC, 3x FM) - *Pseudoramibacter alactolyticus* (1x in 1 case; 8%; 1x FM) - *Bifidobacterium dentium* (3x in 2 cases; 17%; 3x RC) - Mogibacterium timidum (2x in 1 case; 8%; 1x RC, 1x FM) - Filifactor alocis (4x in 3 cases; 25%; 2x RC, 2x FM) - *Shuttleworthia satelles* (2x in 1 case; 8%; 1x RC, 1x FM) - *Lachnoanaerobaculum saburreum* (3x in 1 case; 8%; 1x RC, 2x FM) - *Eggerthia catenaformis* (1x in 1 case; 8%; 1x RC) - *Eubacterium infirmum* (1x in 1 case; 8%; 1x RC) - *Euba yurii* (3x in 2 cases; 17%; 1x RC, 2x FM) - *Atopobium rimae* (5x in 4 cases; 33%; 3x RC, 2x FM) - *Atopobium* spp. (1x in 1 case; 8%; 1x RC) - *Bulleida extructa* (1x in 1 case; 8%; 1x RC) - *Olsenella uli* (17x in 6 cases; 50%; 11x RC, 6x FM) - *Olsenella profusa* (8x in 4 cases; 33%; 3x RC, 5x FM) - *Parascardovia denticolens* (1x in 1 case; 8%; 1x RC) - *Parvimonas micra* (16x in 7 cases; 58%; 10x RC, 6x FM) |

*Supplementary Tab. 2: Overview of the occurrence of the microorganisms detected in the present study:*

*Summarized by occurrence in primary or secondary infection (RC or FM) (Species, that were only found in primary or secondary infections marked* ***bold****)*

|  | **Primary infection**  **(2 cases)** | **Secondary infection**  **(10 cases)** | | |
| --- | --- | --- | --- | --- |
| **Sample** | **RC** | **RC** | | **FM** |
| Micro-organisms isolated | *Actinomyces naeslundii*  ***Actinomyces oris***  *Anaeroglobus geminatus*  *Bifidobacterium dentium*  *Capnocytophaga granulosa*  *Dialister pumosintes* (1x)  *Eubacterium yurii*  *Filifactor alocis*  *Fusobacterium nucleatum*  ***Moraxella osloensis* (1x)**  ***Neisseria bacilliformis* (1x)**  *Olsenella uli*  *Parvimonas micra*  *Porphyromonas gingivalis*  ***Prevotella intermedia* (1x)**  *Prevotella nigrescens*  *Rothia dentocariosa*  *Rothia mucilaginosa*  *Slackia exigua*  *Streptococcus anginosus*  *Streptococcus constellatus*  *Streptococcus oralis*  *Tannerella forsythia*  *Veillonella parvula* | *Actinomyces israelii* (1x)  ***Actinomyces johnsonii* (1x)**  *Actinomyces lingnae* (1x)  ***Actinomyces meyeri* (1x)**  *Actinomyces naeslundii*  ***Actinomyces odontolyticus***  ***Actinomyces oris***  ***Actinomyces* spp. (1x)**  ***Alloprevotella tannerae* (1x)**  *Anaeroglobus geminatus*  ***Atopobium rimae***  ***Atopobium* spp. (1x)**  *Bacteroides* spp. (1x)  *Bifidobacterium dentium* (1x)  ***Bilophila wadsworthia* (1x)**  ***black pigmented bacteria* (1x)**  ***Bulleida extructa* (1x)** *Campylobacter rectus* (1x)  ***Campylobacter showae* (1x)**  ***Candida albicans* (1x)**  ***Capnocytophaga gingivalis* (1x)**  ***Capnocytophaga granulosa* (1x)**  ***Capnocytophaga ochracea***  ***Cardiobacterium hominis* (1x)*Dialister invisus***  ***Eggerthia catenaformis* (1x)**  ***Enterococcus durans* (1x)**  ***Enterococcus faecalis***  ***Eubacterium infirmum* (1x)**  *Eubacterium yurii* (1x)  *Filifactor alocis*  *Fusobacterium nucleatum*  *Fusobacterium periodonticum* (1x)  ***Gemella haemolysans* (1x)**  ***Gemella morbillorum* (1x)**  ***Granulicatella adiacens***  ***Kingella oralis* (1x)**  ***Lachnoanaerobaculum saburreum* (1x)**  *Lactobacillus casei/paracasei* (1x) | *Lactobacillus fermentum* (1x)  *Megasphera micronuciformis* (1x)  ***Mogibacterium timidum* (1x)**  *Neisseria elongate* (1x)  *Neisseria flavescens* (1x)  *Neisseria macacae/mucosa* (1x)  *Neisseria oralis* (1x)  *Neisseria* spp. (1x)  *Olsenella profuse*  *Olsenella uli*  ***Parascardovia denticolens* (1x)**  *Parvimonas micra*  *Porphyromonas gingivalis*  *Prevotella nigrescens* (1x)  ***Prevotella oris* (1x)**  ***Prevotella tannerae* (1x)**  ***Propionibacterium acnes***  ***Propionibacterium avidum* (1x) *Dentiradicibacter hellwigii*** **(1x)**  *Pseudoramibacter alactolyticus* (1x)  ***Raoultella* spp. (1x)**  ***Rothia aeria***  *Rothia dentocariosa*  *Rothia mucilaginosa*  ***Shuttleworthia sattelles* (1x)**  *Slackia exigua*  *Streptococcus anginosus*  *Streptococcus constellatus*  ***Streptococcus gordonii* (1x)**  ***Streptococcus intermedius***  ***Streptococcus mitis***  ***Streptococcus mutans* (1x)**  *Streptococcus oralis*  ***Streptococcus salivarius* (1x)**  ***Streptococcus sanguinis***  *Tannerella forsythia* (1x)  *Veillonella parvula*  *Veillonella* spp. (1x) | *Actinomyces israelii*  *Actinomyces lingnae*  *Actinomyces naeslundii*  *Bacteroides* spp.  *Bifidobacterium dentium*  *Campylobacter rectus*  *Fusobacterium periodonticum*  *Lactobacillus casei/paracasei*  *Lactobacillus fermentum*  *Megasphera micronuciformis*  *Neisseria elongata*  *Prevotella nigrescens*  *Pseudoramibacter alactolyticus*  *Tannerella forsythia*  *Veillonella* spp. |

*Supplementary Fig. 1: Total microbial count (in CFU/ml) and aerobic/facultative anaerobic and anaerobic distribution in samples of primary endodontic treatments; listed per root canal.*

*Supplementary Fig. 2: Relative proportion of individual species in the total microbial count in samples of primary endodontic treatments; listed per root canal; the legend for the color codes for microbial species can be found for all figures in Supplementary Fig 5.*

*Supplementary Fig. 3: Relative proportion of individual species in the total microbial count in samples of the root canals of secondary endodontic treatments; listed per root canal; the legend for the color codes for all figures can be found in Supplementary Fig 5.*

*Supplementary Fig. 4: Relative proportion of individual species in the total microbial count in samples of root canal filling material of secondary endodontic treatments; listed per root canal; the legend for the color codes for all figures can be found in Supplementary Fig 5.*

*Supplementary*
*Fig. 5: Detected microbial species (aerobic and facultative anaerobic blue, anaerobic red; legend for Fig. 3 and 4).*
